# ICTV Virus Taxonomy Profile: *Iflaviridae*

**DOI:** 10.1099/jgv.0.000757

**Published:** 2017-04-06

**Authors:** S. M. Valles, Y. Chen, A. E. Firth, D. M. A. Guérin, Y. Hashimoto, S. Herrero, J. R. de Miranda, E. Ryabov, ICTV Report Consortium

**Affiliations:** ^1^​ United States Department of Agriculture, Agricultural Research Service, Gainesville FL 32608, USA; ^2^​ United States Department of Agriculture, Agricultural Research Service, Beltsville MD 20705, USA; ^3^​ Department of Pathology, University of Cambridge, Cambridge CB2 1QP, UK; ^4^​ Department of Biochemistry and Molecular Biology, University of the Basque Country (EHU), Biophysics Institute (CSIC-UPV/EHU), BÂ° Sarriena S/N, Leioa 48940, Spain; ^5^​ Protein Sciences Corporation, Meriden CT 06450, USA; ^6^​ Department of Genetics, Universitat de ValÃ¨ncia, Burjassot, Spain; ^7^​ Department of Ecology, Swedish University of Agricultural Sciences, Uppsala 750 07, Sweden

**Keywords:** *Iflaviridae*, ICTV Report, Taxonomy

## Abstract

*Iflaviridae* is a family of small non-enveloped viruses with monopartite, positive-stranded RNA genomes of approximately 9â€“11 kilobases. Viruses of all classified species infect arthropod hosts, with the majority infecting insects. Both beneficial and pest insects serve as hosts, and infections can be symptomless (Nilaparvatalugens honeydew virus 1) or cause developmental abnormalities (deformed wing virus), behavioural changes (sacbrood virus) and premature mortality (infectious flacherie virus). The host range has not been examined for most members. The most common route of infection for iflaviruses is the ingestion of virus-contaminated food sources. This is a summary of the International Committee on Taxonomy of Viruses (ICTV) Report on the taxonomy of the *Iflaviridae*, which is available at www.ictv.global/report/iflaviridae.

## Virion

**Fig. 1.**
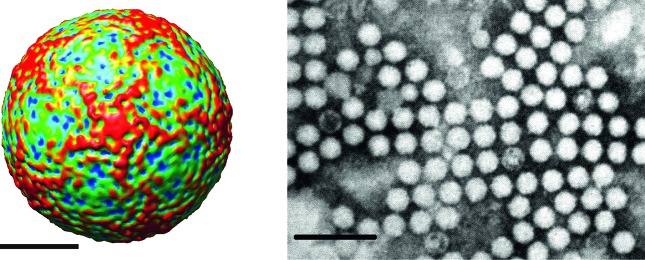
(Left) Surface view of the virion of infectious flacherie virus along a five-fold axis reconstructed by cryo-electron microscopy. The bar represents 10 nm (courtesy of J. Hong). (Right) Negative contrast electron micrograph of the isometric particles of an isolate of infectious flacherie virus. The bar represents 100 nm (courtesy of H. Bando).

Virions are roughly spherical and exhibit icosahedral symmetry with a diameter of 22–30 nm. Virions have no envelope and no distinctive surface structures (Table 1, Fig. 1).

**Table 1.** Characteristics of the family *Iflaviridae*

Typical member:	infectious flacherie virus (AB000906), species *Infectious flacherie virus,* genus *Iflavirus*
Virion	Non-enveloped, 22–30 nm-diameter virions
Genome	9–11 kb of positive-sense, non-segmented RNA
Replication	Cytoplasmic within viral replication complexes formed from a variety of host cellular membranes
Translation	Directly from genomic RNA containing an internal ribosomal entry site (IRES)
Host range	Arthropoda
Taxonomy	Member of the order *Picornavirales*; >10 species in the single genus *Iflavirus*

## Genome

Iflaviruses possess single-stranded, positive-sense, non-segmented RNA genomes with a single open reading frame (ORF). The ORF is translated directly into a polyprotein that is subsequently processed to yield structural, i.e. capsid (N-terminal region) and non-structural (C-terminal region) proteins [1]. The 5′ end of the genome bears a covalently linked protein, VPg, which plays a role in RNA replication.

## Replication

**Fig. 2.**
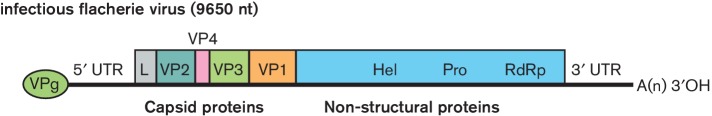
Genome structure of infectious flacherie virus. The genome encodes a single polyprotein that is auto-catalytically cleaved into three major structural proteins (VP1, VP2 and VP3) and non-structural proteins used in replication. The structural proteins are encoded in the 5′-proximal region of the genome and the non-structural proteins are encoded in the 3′-proximal region. The 5′ end of the genome bears a covalently linked protein, VPg, which plays a role in RNA replication.

Replication occurs in the host cell cytoplasm. The coding regions for capsid proteins, arranged in the order VP2–VP4–VP3–VP1, are often preceded by a region encoding a short leader protein (L) of unknown function that is removed from VP2 before capsid assembly. VP4 is analogous to the VP4 present in some dicistroviruses and, in the case of infectious flacherie virus, is present as a minor structural component of the capsid. The non-structural proteins include an RNA helicase, a 3C-like cysteine protease and an RNA-dependent RNA polymerase (Fig. 2). Evidence suggests that translation in some iflaviruses is mediated by an internal ribosomal entry site (IRES) located in the 5′ UTR [2]. The viral RNA is infectious and serves as both genomic and viral mRNA. The mechanisms of polyprotein processing and the effects on host cell macromolecular synthesis during infection have not been well studied for the members of this family.

## Taxonomy

Currently, members of the family are placed together within a single genus, *Iflavirus*. However, phylogenetic analysis of the complete translated genomes of iflaviruses shows that a number of distinct clades are present. These may be separated taxonomically into different genera in the near future as more virus sequences become available.

All member viruses have been isolated from arthropods, primarily insects. Beyond the original host descriptions, the host range of most members has not been examined. However, honeybee iflaviruses, deformed wing virus, Varroa destructor virus 1, slow bee paralysis virus and sacbrood virus have been shown to infect other *Apis* species, as well as several *Bombus* species [3]. Deformed wing virus, Varroa destructor virus 1 and slow bee paralysis virus can also be vectored to honeybees by parasitic mites (*Varroa* and *Tropilaelaps* genera). *Varroa* and *Tropilaelaps* mites are also capable of serving as hosts for deformed wing virus and Varroa destructor virus 1. Deformed wing virus and Varroa destructor virus 1 are also vertically and sexually transmitted in honey bees. The most common route of infection for iflaviruses is the ingestion of virus-contaminated food sources. Trophallaxis in social insects facilitates intra-colonial virus dispersal [4]. In addition to the gut, gonads, fat body, muscle, brain and glandular tissues also have been shown to be a target for several iflaviruses. Once the virus gains entry to the host cell, the infection process is rapid, with progeny virus being produced in hours [5].

## Resources

Full ICTV Online (10th) Report: www.ictv.global/report/iflaviridae.
